# Novel animal model defines genetic contributions for neuron-to-neuron transfer of α-synuclein

**DOI:** 10.1038/s41598-017-07383-6

**Published:** 2017-08-08

**Authors:** Trevor Tyson, Megan Senchuk, Jason F. Cooper, Sonia George, Jeremy M. Van Raamsdonk, Patrik Brundin

**Affiliations:** Center for Neurodegenerative Science, Van Andel Research Institute, Grand Rapids, Michigan USA

## Abstract

Cell-to-cell spreading of misfolded α-synuclein (α-syn) is suggested to contribute to the progression of neuropathology in Parkinson’s disease (PD). Compelling evidence supports the hypothesis that misfolded α-syn transmits from neuron-to-neuron and seeds aggregation of the protein in the recipient cells. Furthermore, α-syn frequently appears to propagate in the brains of PD patients following a stereotypic pattern consistent with progressive spreading along anatomical pathways. We have generated a *C. elegans* model that mirrors this progression and allows us to monitor α-syn neuron-to-neuron transmission in a live animal over its lifespan. We found that modulation of autophagy or exo/endocytosis, affects α-syn transfer. Furthermore, we demonstrate that silencing *C. elegans* orthologs of PD-related genes also increases the accumulation of α-syn. This novel worm model is ideal for screening molecules and genes to identify those that modulate prion-like spreading of α-syn in order to target novel strategies for disease modification in PD and other synucleinopathies.

## Introduction

Parkinson’s disease (PD) is a neurological disorder characterized by the formation of intraneuronal inclusions, Lewy bodies (LB) and Lewy neurites (LN), comprised primarily of α-synuclein (α-syn) and by death of dopamine neurons in the substantia nigra. Several point mutations of the *SNCA* gene (coding for α-syn) as well as duplication and triplication of the gene are associated with familial PD and genome wide association studies have identified single nucleotide polymorphisms around the SNCA locus as risk factors for idiopathic PD^1^. Results from numerous cell culture and animal experiments indicate oligomeric and/or fibrillar forms of α-syn are cytotoxic^2^. Taken together, the evidence that α-syn plays a pivotal role in PD pathogenesis is compelling.

Neuropathological analysis in PD suggests that α-syn pathology first appears in the olfactory system, peripheral nerves and the brainstem, and then progressively involves additional brain regions following a defined pattern, which correlates with the appearance of additional neurological symptoms and signs^3^. Further evidence for the spreading of α-syn pathology within the brain comes from observations of LBs and LNs inside intrastriatal grafts of embryonic nigral tissue that was implanted over a decade prior to analysis^4–8^. It was suggested that the appearance of pathological α-syn aggregates inside the grafted neurons was due to cell-to-cell transfer of α-syn assemblies from host to graft cells, followed by the seeding of further α-syn aggregation. By extension it was proposed that a similar “prion-like” behavior of α-syn along defined anatomical pathways could explain the stereotypic progression of Lewy pathology in the PD brain. Numerous experimental models have now provided convincing support for this prion-like hypothesis of α-syn, i.e. α-syn transfers from cell-to-cell and once inside a new cell can seed the aggregation of endogenous α-syn, triggering the formation of larger assemblies^9–14^.

The mechanisms of α-syn cell-to-cell transfer are largely unknown, however, several proteostasis mechanisms have been suggested to influence α-syn and regulate release and uptake of aberrant α-syn species. These mechanisms are reviewedd in detail elsewhere^15, 16^ and include endo/exocytosis, the lysosomal and autophagy pathway, sorting of endosomal compartments and proteolytic clearance from the extracellular space. The presence of misfolded or aggregated α-syn within the cell can cause the disruption of many of these mechanisms resulting in a vicious cycle of aggregation and propagation. However, the complete set of genes and molecular pathways that contribute to α-syn homeostasis are unknown.

To this end, we have generated a unique *C. elegans* strain that uses bimolecular fluorescence complementation (BiFC) to visualize neuron-to-neuron transfer and dimerization of α-syn^17^. This allows us, for the first time, to monitor interneuronal α-syn propagation in a live animal in real-time and represents a paradigm that is suited for high throughput genetic screens. Using this model, we show that α-syn progressively propagates and accumulates in the cell body and axons of connected neurons as worms age. We also demonstrate that manipulation of worm orthologs of genes associated with inherited PD or pathways linked to PD, such as autophagy influences the rate of α-syn propagation, suggesting that they are crucial to fundamental cellular processes that govern propagation of α-syn pathology.

## Results

### Development of a *C. elegans* model of interneuronal α-syn propagation

**Figure 1:**
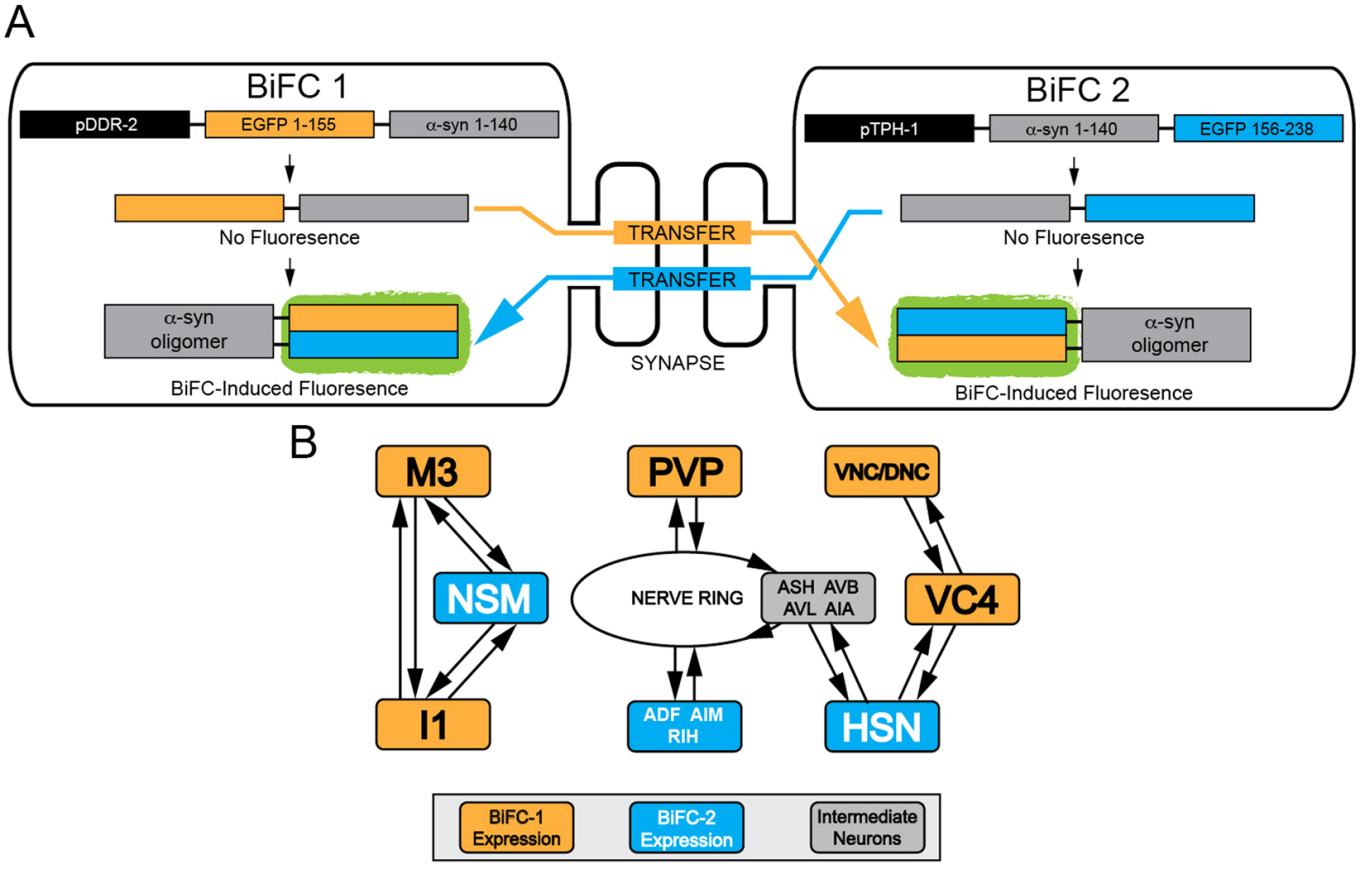
Using BiFC-induced fluorescence to monitor neuron-to-neuron transfer of a-synuclein. (**A**) Model of BiFC-induced fluorescence following transfer of BiFC1 molecules (full-length WT α-syn tagged with N-terminus EGFP) expressed in cells under the control of the *ddr-2* promoter to cells expressing BiFC2 molecules (full-length WT α-syn tagged with C-terminus EGFP) under the control of the *tph-1* promoter, and vice-versa. BiFC1 and BiFC2 molecules combine following α-syn transfer and dimerization resulting in the fluorescence of the now functional EGFP molecule. (**B**) Schematic of some of the neurons involved in cell-to-cell transfer of BiFC-tagged α-syn in our model. Boxes represent BiFC-1-expressing neurons (orange), BiFC-2-expressing neurons (blue) or several non-BiFC expressing intermediate neurons (grey). VNC = Ventral Nerve Cord, DNC = Dorsal Nerve Cord.

**Figure 2:**
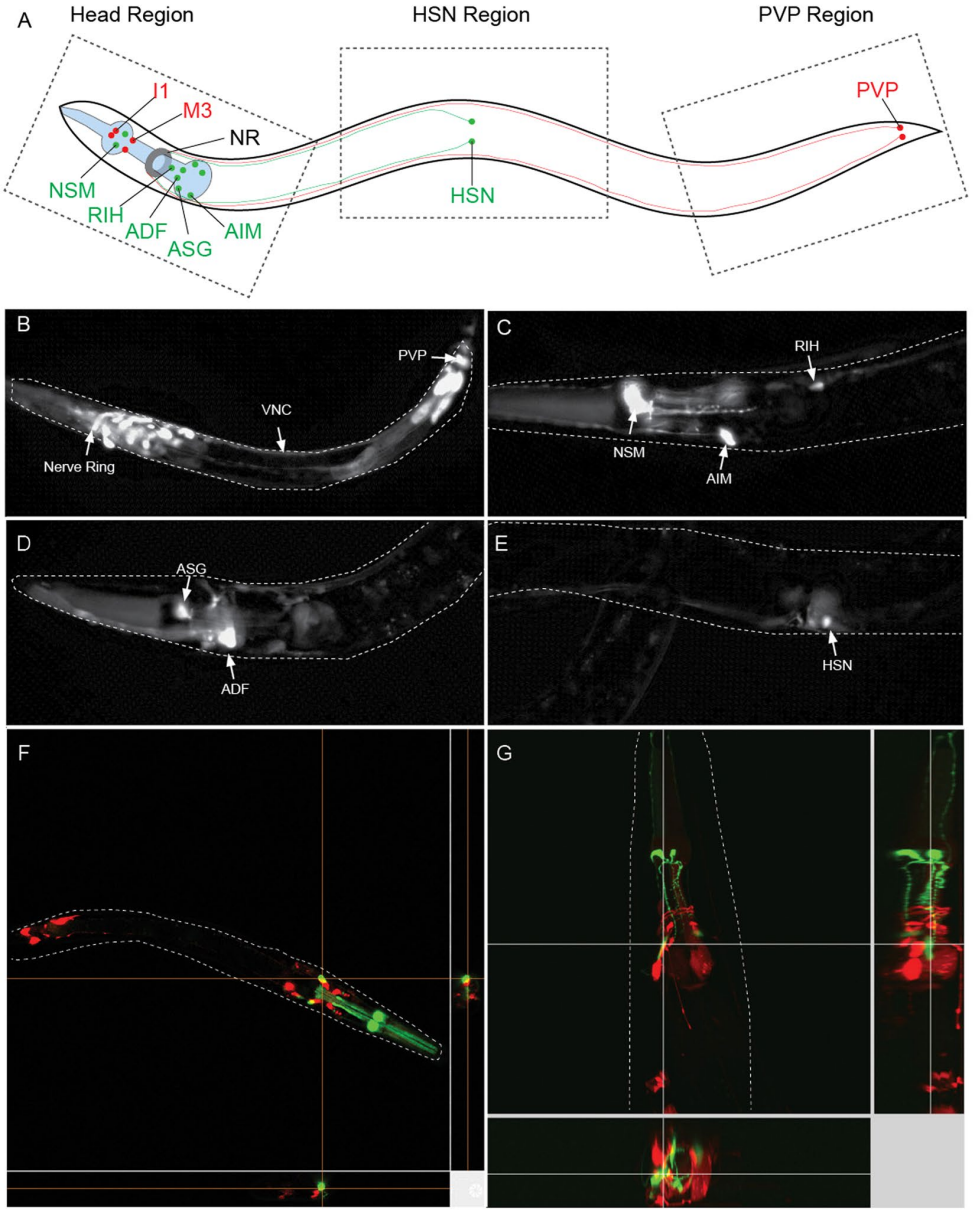
Expression from *ddr-2* and *tph-1* occurs in distinct populations of neurons. (**A**) Location of *ddr-2* expressing neurons (red) and *tph-1* expressing neurons (green), all neurons are bilaterally symmetrical apart from RIH. Neurons depicted with connections in the nerve ring (NR) are ADF, AIM, RIH, HSN and PVP. Not all *ddr-2* expressing neurons with connections to the NR are depicted. (**B**) L4 worm expressing ddr-2::mCherry, expression is evident in neurons in the head region as well as PVP neurons and the ventral nerve cord (VNC). (**C–E**) Adult worms expressing tph-1::mCherry, expression is visible exclusively in serotonergic neurons. (**F–G**) Confocal images of L4 worm (**F**) and the head region of a 1 day old adult worm (**G**) expressing ddr-2::mCherry and tph-1::GFP. Orthogonal view of images confirms there is no co-expression of mCherry or GFP reporters in any neurons. See also supplementary video 1.

In order to visualize α-syn transfer in living worms we utilized BiFC-induced GFP fluorescence. We expressed complementary BiFC-tagged α-syn under the control of the *ddr-2* and *tph-1* promoters (Fig. 1A, Supplementary figure 1). DDR-2 is expressed in neurons in the head and tail of the animal as well as the ventral and dorsal nerve cords^18^. The tryptophan hydroxylase precursor, TPH-1, is expressed solely in the 11 serotonergic neurons present in the hermaphrodite worm^19^. The promoters selected drive expression of these constructs in distinct subsets of neurons, which are synaptically connected (Fig. 1B). The *tph-1* expressing NSM neurons in the head of the worm are directly connected to the *ddr-2* driven M3 and I1 neurons. Most of the other neurons involved share synaptic connections in the nerve ring of the worm, these include the *tph-1-*expressing HSN neurons and the *ddr-2-*expressing PVP neurons located at the vulva and the tail of the worm respectively (Figs 1B and 2A). We confirmed α-syn protein expression by Western blot (Supplementary figure 3) and estimated to be present at 12.5 ng/mg of total protein.

Promoter specificity was determined by placing mCherry under the control of *ddr-2* and *tph-1* promoters in separate strains (Fig. 2B–E). These strains confirmed expression in the expected neuronal populations (Fig. 2C–E). To confirm that the expression of both promoters is exclusive to these specific sub-populations, we generated a double transgenic worm expressing GFP in *tph-1* neurons and mCherry in *ddr-2* neurons. Confocal imaging of these worms showed that no neurons co-expressed mCherry and GFP, in larval or adult worms (Fig. 2F,G, Supplementary Video 1). Thus, not only do these promoters induce expression in distinct neurons but GFP alone does not transfer to mCherry-expressing neurons, or vice versa, at detectable levels. Furthermore, control worms expressing the complementary BiFC tags without α-syn under the control of the same promoters did not produce any fluorescence up to 15 days of age (Supplementary figures 1, 2). Therefore, any EGFP expression observed in this model can only occur following the transfer of α-syn from one neuron to another, followed by dimerization of the α-syn tagged with a complementary truncated EGFP molecule.

### Progressive accumulation of α-syn in neurons and axons

**Figure 3:**
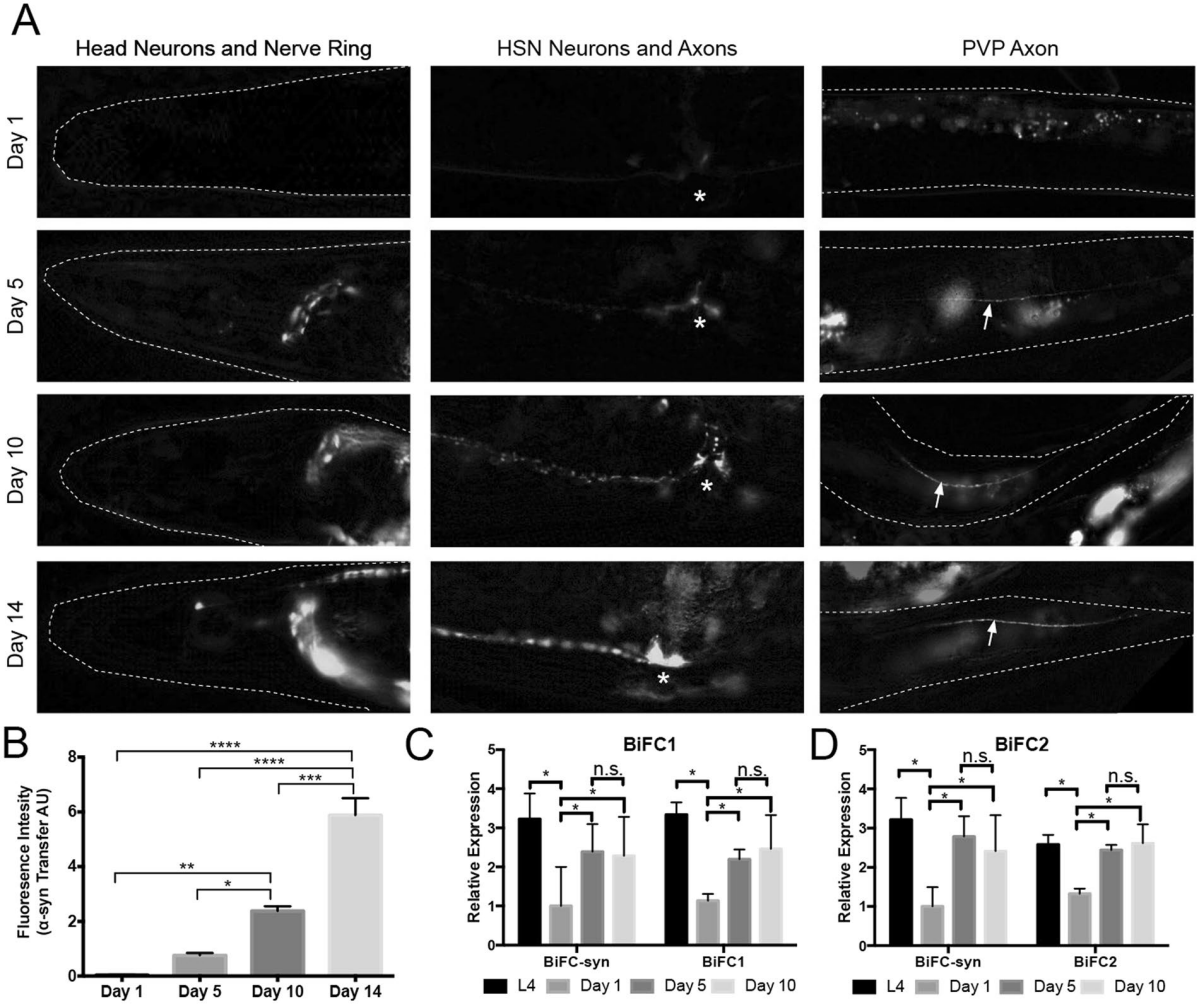
BiFC-syn *C. elegans* model reveals neuron-to-neuron transfer that increases with age. (**A**) BiFC-induced expression of EGFP detected in the head region and nerve ring, the HSN neurons and axon and the PVP axon in the tail region of the worm in 1, 5, 10 and 14 days old BiFC-syn worms. * Denotes location of vulva. (**B**) Quantification of the fluorescence intensity in the nerve ring and head region of 1, 5, 10 and 14 days old worms. Statistical significance was determined from the mean values of three independent experiments with at least 75 worms per group, using ANOVA in conjunction with a Tukey’s post-hoc multiple comparisons test (alpha = 0.05) (**C**) Relative mRNA levels of the BiFC1 construct in BiFC-syn worms and control worms expressing only the BiFC1 construct in L4, 1, 5 and 10 days old worms. (**D**) Relative mRNA levels of the BiFC2 construct in BiFC-syn worms and control worms expressing only the BiFC2 construct in L4, 1, 5 and 10 days old worms. Statistical significance was determined from the mean values of three independent qPCR experiments using ANOVA in conjunction with a Tukey’s post-hoc multiple comparisons test (alpha = 0.05). In all cases data is represented as mean of 3 independent experiment, represented as mean ± SEM. See also supplementary video 2.

We monitored EGFP fluorescence in BiFC-syn worms of different ages to evaluate α-syn transfer and subsequent dimerization. In larval worms, we initially observed EGFP fluorescence in the mini-network consisting of the BiFC1-expressing M3 and I1 neurons and BiFC-2 expressing NSM neurons in the head region of the worm (Fig. 1B, Supplementary Video 1). This fluorescence decreased in one day-old worms, likely due to a combination of protein clearance in these cells and a drop in BiFC mRNA expression levels (Fig. 3C,D). However, low levels of fluorescence were still evident in one day-old adult worms. This was most notable in the nerve ring, NSM neurons and HSN neurons (Fig. 3A, Supplementary Video 1). By day 5 of adulthood, fluorescence in the nerve ring significantly increased (Fig. 3A,B, Supplementary video 2), fluorescence also increased in the HSN neurons and axons HSN and had spread to the PVP axons (Fig. 3A).

This pattern of progression continued with fluorescence of gradually increasing intensity developing in all connected neurons and axons in 10 to 14 day-old worms (Fig. 3A, Supplementary Video 2). The most notable increase in EGFP fluorescence intensity occurred in the nerve ring of the worm, where the majority of BiFC1 and BiFC2-expressing neurons share synaptic connections. By day 14, fluorescence was also clearly seen in the dorsal and ventral nerve cords (Supplementary Video 2).

Quantification of the fluorescence intensity in the head region of the worm at day 1, 5, 10 and 14 days post-adulthood indicated a significant increase of α-syn accumulation as the worms aged (Fig. 3B). Importantly, mRNA expression of the BiFC1 and BiFC2 constructs remained relatively unchanged from day 5 to day 10 (Fig. 3C,D), therefore, the increase in fluorescence cannot be attributed to increased expression of the transgenes.

### Synaptic transmission influences α-syn propagation

As α-syn is present at high levels at the synapse, has been associated with neurotransmitter release and transfers along interconnected brain regions *in vivo* ^20, 21^, the synapse is the most like point of transmission of α-syn. To test whether modulation of synaptic vesicle release would affect the rate of α-syn propagation in BiFC-syn worms, we used two mutant strains, *tom-1* and *unc-11*. The *tom-1* mutant strain exhibits prolonged synaptic vesicle release^22^. This is also associated with an increase in the number of plasma membrane-contacting vesicles in tom-1 mutant synapses. Conversely, the *unc-11* mutant strain exhibits decreased synaptic vesicle release due to the mislocalization of synaptobrevin disrupting SNARE complex formation^23^.

**Figure 4:**
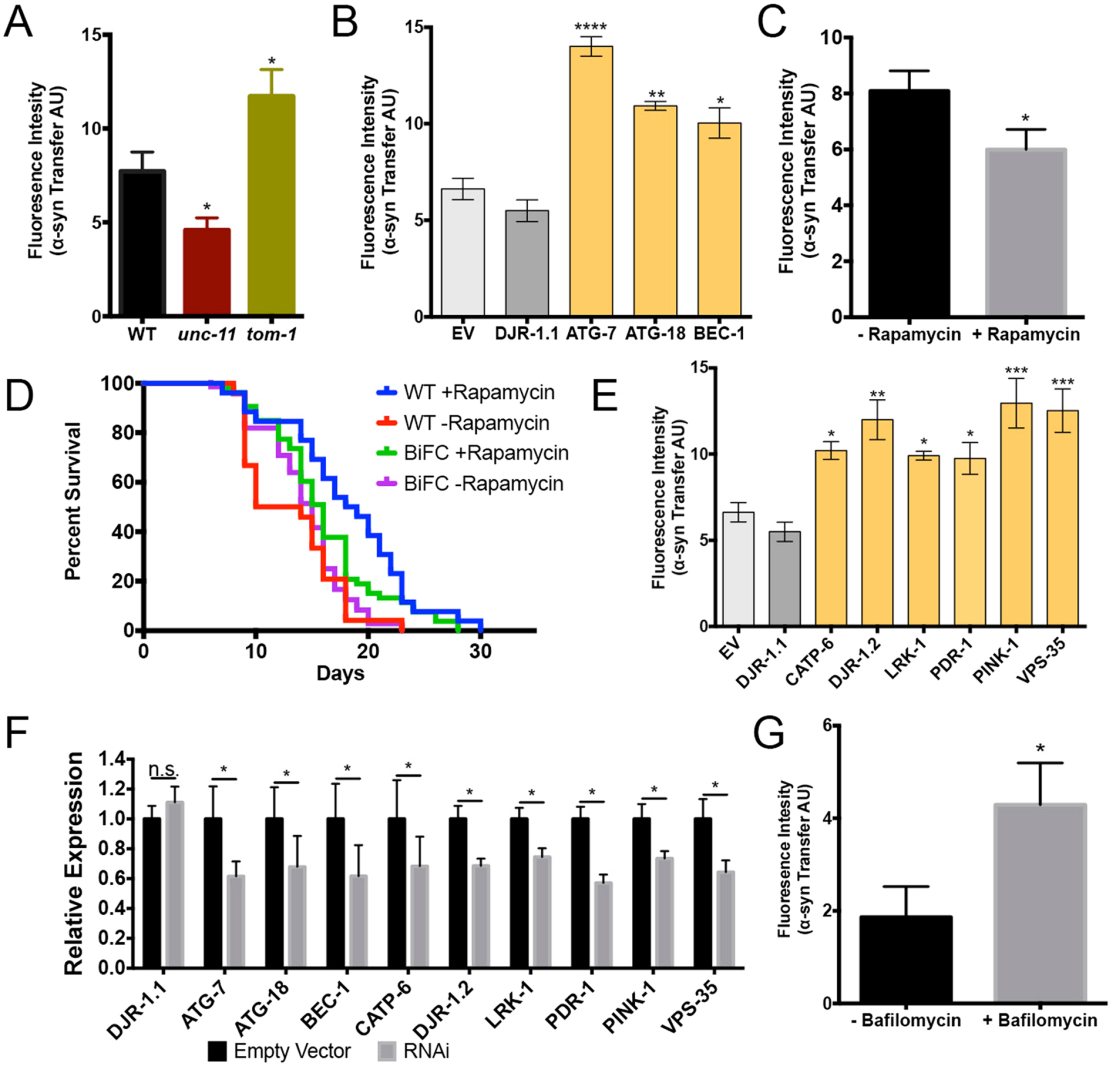
Neuron-to-neuron transfer of a-syn requires synaptic transmission and is increased by disruption of autophagy or PD-associated genes. (**A**) Quantification of BiFC-induced fluorescence in BiFC-syn (WT) worms compared to BiFC-syn worms crossed with *unc-11* and *tom-1* mutant strains. (**B**) Quantification of BiFC-induced fluorescence in BiFC-syn worms crossed into the neuronal RNAi sensitive TU3401 strain following RNAi by feeding on HT115 bacteria expressing dsRNA of the respective autophagy related genes of interest. EV: Empty Vector control (light grey), DJR-1.1: off-target control, *C. elegans* isoform of DJR-1 expressed primarily in intestine (dark grey). (**C**) Quantification of BiFC-induced fluorescence in the head region of the worm of BiFC-syn worms following rapamycin treatment. (**D**) Lifespan of N2 and BiFC-syn worms following rapamycin treatment and without. (**E**) Quantification of BiFC-induced fluorescence in BiFC-syn worms crossed into the neuronal RNAi sensitive TU3401 strain following RNAi by feeding on HT115 bacteria expressing dsRNA of the respective autophagy related genes of interest. EV: Empty Vector control (light grey), DJR-1.1: off-target control. (**G**) Quantification of BiFC-induced fluorescence in the head region of the worm of BiFC-syn worms following bafilomycin treatment. (**A–C,E,G**) Statistical significance was determined from the mean values of three independent experiments with at least 75 worms per group, using ANOVA in conjunction with a Tukey’s post-hoc multiple comparisons test (alpha = 0.05). (**F**) mRNA expression levels of genes following RNAi knockdown. Statistical significance was determined from the mean values of three independent qPCR experiments using ANOVA in conjunction with a Tukey’s post-hoc multiple comparisons test (alpha = 0.05). In all cases data is represented as mean of 3 independent experiment, represented as mean ± SEM.

We crossed BiFC-syn worms into both of these mutant strains independently. We then allowed the worms to age for 10 days and measured the fluorescence intensity in the head region of the worms as described above. We found that *tom-1* mutants consistently exhibited significantly more fluorescence compared to BiFC-syn WT worms (Fig. 4A). On the other hand, *unc-11* mutants showed significantly less BiFC-induced fluorescence compared to controls. Taken together, these observations suggest that transfer of α-syn is linked to synaptic vesicle release and therefore key exocytic and endocytic pathways are likely to play a role in neuron-to-neuron α-syn transfer.

### Autophagy and PD-related genes influences α-syn accumulation and transfer

The role of autophagy has been studied extensively in α-syn turnover and accumulation. Timely clearance of aberrant α-syn species is crucial in order to prevent the spread of α-syn aggregates. Aggregation-prone α-syn which is not degraded eventually seeds misfolding and aggregation of normal synuclein in the cell^24^, which can further promote cell-to-cell spreading of pathology. We knocked down autophagy genes in BiFC-syn worms crossed into a strain in which RNAi is only possible in neuronal cells, allowing us to silence genes that would otherwise cause detrimental phenotypes^25^. We targeted the *C. elegans* genes, *atg-7*, *atg-18* and *bec-1*, which are involved in autophagic function at different stages of the process through vesicle expansion, vesicle recycling and vesicle nucleation, respectively^26^. We then monitored the fluorescence intensity in the nerve ring and head neurons of BiFC-syn worms and found that it was significantly increased in 10 day-old BiFC-syn worms compared to age-matched controls (Fig. 4B). This suggests that the disruption of autophagy leads to a net increase in α-syn accumulation over time.

The mTOR inhibitor rapamycin results in an induction of autophagy, leading to, e.g., longer lifespan in worms^27^. In our hands, induction of autophagy by rapamycin led to a decrease in α-syn accumulation in BiFC-syn worms and a modest increase in lifespan of BiFC-syn and wild-type (N2) worms (Fig. 4C,D). Conversely, inhibition of autophagy with bafilomycin A1 resulted in a two-fold increase in α-syn transmission when fed to L1 larvae in liquid culture for 72 hours (Fig. 4G). These findings are consistent with our other observations and add further weight to the idea that autophagy plays a key role in cellular handling of α-syn.

The orthologs of some PD-related genes are present in *C. elegans*, some of which have previously been shown to influence α-syn induced phenotypes^28, 29^. We knocked down the expression of the PD-related genes; *catp-6 (atp13a2/park-9)*, *djr-1.2* (*dj-1*), *lrk-1* (*lrrk2*), *pdr-1* (*parkin/park-2*) and the retromer associated *vps-35* gene and quantified the amount of transfer. Interestingly, we found that the knockdown of each of these PD-related genes, individually, in BiFC-syn worms caused an increase in fluorescence as assessed in 10 day-old worms (Fig. 4E). Surprisingly, the knockdown of other retromer associated proteins, sorting nexins *snx-1*, *snx-6* and *snx-27* did not affect the rate of α-syn transmission (Supplementary figure 4). These results suggest that these PD-related genes interact with molecular pathways that influence the transmission of α-syn from neuron to neuron, at some level of the process.

## Discussion

Many publications have addressed interneuronal propagation of α-syn pathology in mammalian models^9–14, 30^ However, due to the nature (technical and temporal limitations, expense, etc.) of these mammalian models, those studies have typically focused on the role of a single aspect of this complex process. A recent study has also used *C. elegans* to show that α-syn cell-to-cell transfer increases due to aging-related genetic variations^31^. However, this model focuses on transfer from pharyngeal muscle cells and neurons that innervate the pharynx. While it is clear that neuromuscular junctions might share some endocytic and exocytic processes with interneuronal synapses, the manner in which they handle internalized α-syn may be different to that of neuronal cells as it is known that endocytosis, exocytosis and autophagy are all closely coupled to neural innervation of muscle^32^.

Here, we have generated a novel quantitative *C. elegans* system that specifically assays neuron-to-neuron transfer and dimerization of α-syn, modelling the transfer of α-syn throughout interconnected regions in the human brain^13, 33^. This model provides a unique and powerful tool suitable for unbiased screening of genes using siRNA libraries and/or small-molecule compounds that influence α-syn interneuronal transfer. To demonstrate the validity of this model we targeted two key pathways; the endocytosis/exocytosis pathway and the lysosomal-autophagy system. In both cases we found that disruption of the pathway influenced the rate at which α-syn transferred and accumulated in neurons. We also found that silencing the worm orthologs of six key PD-related genes caused an increase in the accumulation of α-syn in neurons, lending further insight into how these genes interact with α-syn and their role in the pathophysiology of PD.

Our primary observation was that BiFC-induced fluorescence, caused by the transfer and dimerization of α-syn, accumulates over time in synaptically connected neurons and axons in the worm. The emergence of fluorescence in increasing numbers of neurons in the worm over time is analogous to the cell-to-cell spread of α-syn pathology that has been suggested to occur in the PD brain, albeit on an anatomically smaller scale and a very compressed time line. Furthermore, since dimerization of α-syn must occur in order to induce fluorescence, even if the initial dimers are beyond our limits of detection, we can establish that the first step(s) of the α-syn aggregation process occurs. An important caveat of this model is that the BiFC tags may prevent higher order oligomers from forming and we did not explore if higher order oligomers of α-syn are formed in this system. Furthermore, any α-syn aggregation that might occur will be influenced by the presence of the BiFC tags and may not fully represent the aggregation that is seen in PD. Nevertheless, it is still clear, that in our model, α-syn transfers from cell-to-cell and that it accumulates within these cells over time.

Our data suggest that α-syn is transferred at the synapse during neurotransmission. By crossing BiFC-syn worms into *tom-1* and *unc-11* mutants, we show that the rate of α-syn accumulation in BiFC-syn worms is related to regulation of synaptic vesicle release and uptake. It is believed that α-syn is involved in maintaining neurotransmitter homeostasis by regulating synaptic vesicle fusion, clustering, and trafficking and interacting with neurotransmitter membrane transporters in mammalian systems^34^. As these systems are evolutionarily conserved, it is likely that exogenous α-syn will interact with these mechanisms in *C. elegans*. This would acquiesce with studies showing that α-syn transfers between interconnected brain regions^33^ and suggests that potentially, the synapse is a key site of α-syn propagation and pathology.

In this model we also show that the inhibition of autophagy, by RNAi knockdown of key autophagy genes or by the use of the autophagy inhibitor bafilomycin, causes an increase in the intercellular exchange and dimerization of α-syn over time within specific neurons. In a separate experiment, we also show that α-syn dimerization is reduced when autophagy is induced through the use of rapamycin. Protein clearance mechanisms play a crucial role in α-syn proteostasis. Higher order oligomeric α-syn species are degraded by macroautophagy while monomeric and dimeric species are degraded by chaperone mediated autophagy or by the ubiquitin proteasome system^35^. However, it has been established that aggregation prone mutants of α-syn, over-expression of α-syn and LB-like α-syn inclusions inhibit autophagy, resulting in a vicious cycle where α-syn degradation is impaired, leading to larger aggregates and eventually cell death^36^. Furthermore, in order to maintain cellular homeostasis, the inhibition of autophagy also leads to an increase in exocytosis^37–39^. Similar observations in our worm model strengthen these arguments and further validate the autophagy/lysosomal pathway as a major factor in the transmission of α-syn. As we have shown earlier, the regulation of synaptic vesicle release effects α-syn transmission. It is therefore likely that inhibition of autophagy leads to increased synaptic vesicle release in affected neurons. Whether or not this does indeed occur as a consequence of reduced autophagy in this model is not known, but warrants further investigation.

As we have discussed in depth elsewhere^15, 40^, autophagy, aggregation of α-syn, and protein uptake and release are closely intertwined. It therefore comes as no surprise that many PD-related genes have been shown to effect one or more of these processes. Using our BiFC-syn model in conjunction with RNAi to knockdown PD-related genes resulted in an increase of α-syn neuron-to-neuron transfer and accumulation in each case. This observation serves to highlight the complexity of the mechanisms involved and how these genes effect downstream processes, eventually contributing to pathology.

Of the six PD-related genes that we targeted, several have been directly linked to the autophagy/lysosomal pathway; mutations in *catp-6* (*atp13a2*) and *lrk-1* (*lrrk2*) have been shown to impair autophagy^41–43^, while mutant *vps-35* impairs the ability of the retromer to deliver essential lysosomal enzymes, impacting lysosomal function^44^. Interestingly, knockdown of the sorting nexins; *snx-1*, *snx-6* or *snx-27*, did not have any effect on the transmission of α-syn suggesting that either there is some redundancy in which sorting nexins are recruited to the retromer or that *vps-35* has some other function unrelated to the retromer.

Others; *djr-1.2* (dj-1), *pink-1* and *pdr-1* (*parkin*), as well as *atp13a2*, are important genes in the regulation of reactive oxygen species (ROS) and mitochondrial health^45, 46^. The regulation of oxidative stress plays a crucial role in PD pathology in that the aggregation of α-syn causes an increase in ROS and increased ROS within the cell induces aggregation of α-syn, resulting in a feedback loop^47, 48^, eventually contributing to autophagic failure, α-syn release and cell death as discussed earlier.

Of course, there is some overlap in the processes that these genes are involved in. LRRK2, for instance, is a large, multifunctional protein involved in vesicular trafficking, microtubule/cytoskeletal network formation, synaptogenesis and mRNA translation, all of which may contribute to PD pathology and α-syn spread^49^. *Parkin* has also previously been shown to be involved in lipid raft-dependent endocytosis and cell-to-cell transmission of α-syn^50^, while mutant *Parkin* leads to increased generation of intraluminal vesicles and greater release of exosomes^51^.

The emerging picture is that multiple mechanisms can influence the propagation of α-syn from neuron to neuron. The model system that we present here reveals that modulation of endo/exocytosis, autophagy and PD-related genes that are known to be involved in these mechanisms alter α-syn transfer and accumulation in connected neurons. However, the complete set of processes that contribute to transmission of α-syn are still not known and novel mechanisms or proteins are likely to be involved. For example, α-syn transmission was recently shown to be initiated by binding to lymphocyte-activation gene 3 (LAG3) in human dopaminergic neurons^52^. This BiFC-syn worm model can readily be applied to whole genome RNAi screens to discover novel modifiers of α-syn interneuronal transfer. Additionally, this model would be equally amenable to test novel drugs and compounds for their ability to effect α-syn transfer. The existence of automated technologies that monitor fluorescence in *C. elegans* in high-throughput fashion makes this approach feasible^53–55^. In turn, knowledge derived from such screens can be applied to develop therapeutic strategies that modify propagation of α-syn pathology, and potentially slow PD progression.

## Materials and Methods

### Worm Strains and Maintenance

*C. elegans* strains were cultured on NGM medium and maintained at 20 °C unless otherwise stated using standard techniques. Strains N2 (wild-type), TU3401 [*sid-1(pk3321*) V; *uIs69[pCFJ90(myo-2p::mCherry) + unc-119p::sid-1]* V] (maintained at 16 °C) and VC223 (*tom-1(ok285)* I) were obtained from the Caenorhabditis Genetics Center, which is funded by NIH Office of Research Infrastructure Programs (P40 OD010440). Strain tm5447 (*unc-11(e47)* I) was obtained from the National BioResource Project, Tokyo, Japan, which is part of the International *C. elegans* Gene Knockout Consortium^56^.

### Plasmid Construction

Plasmids containing BiFC-tagged α-syn (EGFH1-LINK-SYN & SYN-EGFH2) were provided by Dr. Tiago Outeiro^17^. These were used as template to generate worm expression plasmids as follows. The *tph-1* promoter (1.8 Kb upstream from start codon) was amplified from genomic *C. elegans* N2 DNA using primers designed to add Gateway attB4 and attB1 sequences (forward primer: ggggacaactttgtatagaaaagttgcgtgtaccctgaccaaaaccaatac, reverse primer: ggggactgcttttttgtacaaacttggatgattgaagagagcaatgcta). The BiFC1 and BiFC2 fragments were amplified from EGFH1-LINK-SYN and SYN-EGFH2 respectively using primers designed to add Gateway attB1 and attB2 sequences (BiFC1; forward primer: ggggacaagtttgtacaaaaaagcaggctggatggtgagcaagggcgagg, reverse primer: ggggaccactttgtacaagaaagctgggtgttaggcttcaggttcgtagtc. BiFC2; forward primer: ggggacaagtttgtacaaaaaagcaggctggatggatgtattcatgaaaggac, reverse primer: ggggaccactttgtacaagaaagctgggtgttacttgtacagctcgtccatg). The *C. elegans* unc-54 3′ UTR was amplified from N2 genomic DNA using primers designed to add the Gateway attB2r and attB3 sequences (forward primer: ggggacagctttcttgtacaaagtgggggtccaattactcttcaacatc, reverse primer: ggggacaactttgtataataaagttggtgcggtcataaactgaaacg). These individual amplicons were recombined with the Gateway plasmids pDONR 221 P4-P1r, pDONR 221 & pDONR 221 P2r-P3, respectively in a BP reaction. These were then inserted into the PCG150 plasmid (Addgene) by performing an LR reaction to generate the pTPH-1-BiFC1 and pTPH-1-BiFC2 worm expression plasmids. mCherry and GFP entry clones were also used in conjunction with the above schema to create pTPH-1-mCherry/GFP worm expression plasmids. To generate pDDR2-BiFC1 and pDDR2-BiFC2 plasmids, the DDR-2 promoter sequence (1.5 Kb from start codon) was amplified from genomic DNA using primers designed to add SmaI and AgeI restriction sites to the amplicon (forward primer: gggaaacccgggggtactaattcccactgaacctg, reverse primer: ccctttaccggtcctgacatagatgagcgt). This was spliced into the cloning site of pPD95.75 (Addgene) to generate a pDDR-2-mCherry worm expression plasmid. The mCherry sequence was cut from this plasmid using AgeI and EcoRI and replaced with the BiFC1 or BiFC2 fragment, amplified from EGFH1-LINK-SYN or SYN-EGFH2 respectively with AgeI and EcoRI restriction sites added (BiFC1; forward primer: gggaaaccggtaatggtgagcaagggcgaggag, reverse primer: ccctttgaattcttaggcttcaggttcgtag. BiFC2; forward primer: ggagggtaccggtaatggatgtattcatgaaag, reverse primer: attccagaattcttacttgtacagctcgtcca) to generate pDDR2-BiFC1 and pDDR2-BiFC2 worm expression plasmids.

pDDR-2-Ctrl and pTPH-1-Ctrl were generated from pDDR-2-BiFC-1 and pTPH-1-BiFC2 by PCR with primers that added a unique cutting site towards the 5′ end of the α-syn cDNA sequence (pDDR2-BiFC1; forward primer: gggcccccatggacgctaccattaccaacttgtctg, reverse primer: gggcccccatggaatacatccttaagggagcctccc, pTPH-1-BiFC-2; forward primer: gggcccggtaccaagtcctttcatgaatacatccat, reverse primer: gggcccggtaccttcgagaagaacggcatcaaggtga). After PCR, amplicons were cut with NcoI and KpnI respectively, annealed and cloned.

### Generation of Transgenic Worms

Transgenic strains were generated by injection of worm expression plasmids into the gonads of N2 *C. elegans* along with the marker *rol-6(su1006)*. Stable transgenic lines were generated that express BiFC1 (EGFH1-LINK-SYN) under the control of the *ddr-2* promoter and BiFC2 (SYN-EGFH2) under the control of the *tph-1* promoter JVR406 [P_*ddr-2*_::BiFC1; P_*tph-1*_::BIFC2; *rol-6(su1006)*]. Worms exclusively expressing either P_*ddr-2*_::BiFC1, P_*ddr-2*_::BiFC2, P_*tph-1*_::BIFC1 or P_*tph-1*_::BIFC2 were also generated by the same method. As were strains JVR402 [P_*tph-1*_::mCherry], JVR412 [P_*tph-1*_::GFP], JVR403[P_*ddr-2*_::mCherry] and JVR401[P_*tph-1*_::GFP; P_*ddr-2*_::mCherry]. Additional strains were generated by crossing with strains TU3401, VC223 and tm5447.

### Confocal Imaging

Worms were mounted on 2% agarose slides in 3 mM levamisole and analyzed on an inverted confocal laser microscope Nikon Eclipse Ti-E (Nikon, USA). All images were generated using NIS Elements AR 4.00.08 software (Nikon, USA).

### Bimolecular Fluorescence Assay

Worm strain JVR406[P_*ddr-2*_::BiFC1; P_*tph-1*_::BIFC2; *rol-6(su1006)*] were grown to L4 stage before being transferred to plates supplemented with 50 uM FUDR and incubated at 20 °C. At the desired time-point worms were mounted on an agarose pad with 3 mM levamisole and the pharyngeal region was imaged at 20x magnification on a fluorescence microscope. Images were analyzed using ImageJ to determine the amount of BiFC-induced GFP expression present in the neurons and their axons in this region. At least 75 worms were analyzed per group and results are expressed as the average of three independent experiments. Statistical significance was determined using ANOVA and Tukey’s post-hoc test.

### RNAi

In order to perform RNAi, specifically in neurons, JVR406 was crossed into strain TU3401 [*sid-1(pk3321)* V; *uIs69[pCFJ90(myo-2p::mCherry)* + *unc-119p::sid-1]* V]. Bacterial RNAi clones were obtained from the Ahringer RNAi library (Source Bioscience). NGM plates were supplemented with 100 µg/ml carbenicillin and 1 mM IPTG. Bacterial clones were grown overnight in LB with 100 ug/ml carbenicillin. The following day, IPTG was added to a final concentration of 1 mM and the bacteria were allowed to grow (shaking at 37 °C) for another hour. 50 µl of culture was seeded onto each plate. One day old adult worms were added to the plate and allowed to lay eggs for 24 hours. 48 hours later, L4s were transferred on to new NGM plates supplemented with with 100 µg/ml carbenicillin, 1 mM IPTG and 50 mM FUDR that were pre-seeded with bacterial RNAi clones. Worms were aged for 10 days on these plates before being examined for BiFC-induced fluorescence as above.

### Quantitative PCR

Knockdown of mRNA levels by RNAi was determined using RT-PCR. cDNA was made from five individual worms (for each biological replicate) using the CellsDirect^TM^ One-Step qRT-PCR Kit (Invitrogen). qPCR was performed with Power SYBR green from Applied Biosystems and cycled on an Applied Biosystems Step One Plus system. All reactions were repeated multiple times: two technical replicates for each of three biological replicates were performed and all data was normalized to *act-1*.

### Rapamycin Treatment

Worms were grown on NGM plates supplemented with 100 uM rapamycin until L4 stage and then moved to plates supplemented with 100 uM rapamycin and 50 uM FUDR for 10 days. Worms were then mounted on to an agarose pad with 3 mM levamisole and microscopically analyzed as above. Control worms were grown on plates containing appropriate concentration of the rapamycin vehicle, DMSO.

### Bafilomycin Treatment

L1 BiFC-syn larvae were synchronized by treating gravid adults with sodium hypochlorite and 10 M NaOH. Released embryos were allowed to hatch onto non-seeded NGM plates for 24 hours. L1s were then washed off the plate in M9 medium and counted. Approximately 100 worms were added to individual wells of a 96-well plate and treated with 100 ug/ml bafilomycin A1 or vehicle (DMSO). OP50 bacteria was added as a food source at a final concentration of 5 mg/ml. Worms were incubated at 20 °C for 72 hours before mounting on to an agarose pad with 3 mM levamisole and microscopically analyzed as before.

### Western Blot

BiFC-syn worms were age synchronized and grown to one day post adulthood on standard NGM plates with OP50. Worms were washed from the plates into modified radioimmunoprecipitation (RIPA) assay buffer containing protease and phosphatase inhibitor mixture and flash frozen in liquid nitrogen. Worms were sonicated for 30 cycles (30 s on, 30 s off) using the Diagenode Bioruptor Sonicator. Protein levels were determined using a BCA kit (Thermo Scientific). BiFC-Syn total lysate (125 μg protein) were mixed with SDS- PAGE sample buffer (to final concentration of 2% SDS, 25 mM Tris–HCl pH 6.8, 1% β-mercaptoethanol). Standards were a serial dilution of recombinant human alpha-synuclein protein (ab51189, Abcam). Samples were heated at 95 °C for 5 min then electrophoresed on 4–20% Tris–tricine SDS–polyacrylamide gels and electro-transferred to nitrocellulose membrane (BioRad Laboratories). To block nonspecific binding sites, membranes were incubated with TBS with 5% skim milk powder followed by antibody incubation with purified anti-α-Synuclein 103–108 Antibody (1:500, BioLegend), followed by HRP-conjugated secondary antibody (Cell Signaling). Signals were detected by chemiluminescence (Thermo Scientific) using a BioRad Imager.

## Electronic supplementary material

Supplementary Information

Supplementary Video 1

Supplementary Video 2
