# Supplementary material for: Novel animal model defines genetic contributions for neuron-to-neuron transfer of α-synuclein
Source: Sci Rep. 2017 Aug 8;7:7506. doi: 10.1038/s41598-017-07383-6 (PMC5548897; doi:10.1038/s41598-017-07383-6)

Novel animal model defines genetic contributions for neuron-to-neuron transfer of α-synuclein

Trevor Tyson, Megan Senchuk, Jason Cooper, Sonia George, Jeremy M. Van Raamsdonk, Patrik Brundin

Supplementary Information

Supplementary Video 1. Confirmation of promoter specificity. Confocal 3D projection movie of worms co-expressing pDDR-2:mCherry and pTPH-1:GFP. Larval worms, and adult worms up to 10 days old were imaged and show no co-expression in any cells or neuronal processes, confirming that these promoters are specific to two anatomically separate subsets of neurons.

Supplementary Video 2. Progressive spreading and accumulation of α-syn in BiFC-syn worms. Confocal 3D projection movie of BiFC-tagged α-syn worms showing the accumulation of α-syn within neurons, following transfer, in the pharyngeal region of the worm at increasing time points. In larval worms, α-syn is immediately seen in the NSM neurons, which increases in one day old worms. By day 5 α-syn is noticeable in the nerve ring of the worm and to a lesser extent, in the dorsal and ventral nerve cord. The level of α-syn significantly increases in 10 day old and again in 14 day old worms.

Supplementary Figure 1. DNA and translated AA of plasmids used to generate BiFC-syn and BiFC-Ctrl worms. A. pTPH-1-BiFC2, B. pTPH-1-Ctrl, C. pDDR-2-BiFC1 and D. pDDR-2-Ctrl.

Supplementary Figure 2. BiFC tags require α-syn to produce fluorescence. BiFC-Ctrl-6 worms expressing BiFC tags with partial α-syn do not produce any fluorescence within neurons or axons at A. L4 stage, B. Day 1, C. Day 5, D. Day 10 or E. Day 15 post adulthood. F. qPCR of several lines of BiFC-Ctrl worms that were generated with BiFC-syn worms (JVR406) and wild-type (N2) probed with specific probes for BiFC1 and BiFC2. Generated line BiFC-Ctrl-6 expresses both constructs at levels similar to BiFC-syn worms.

Supplementary Figure 3. A. Western blot and B. Overexposed Western blot of one day old BiFC-Syn worms total protein lysate (125 ng) with recombinant human α-syn protein standards at increasing amounts (left to right: 0.5 ng, 1 ng, 2 ng, 4 ng, 8 ng, 16 ng, 32 ng, 64 ng). Using regression analysis, the estimated concentration of α-syn is estimated to be 12.5 ng/mg total of total protein. Statistical significance was determined from the mean values of three independent qPCR experiments using ANOVA in conjunction with a Tukey's post-hoc multiple comparisons test (alpha = 0.05).

Supplementary Figure 4. Quantification of BiFC-induced fluorescence in BiFC-syn worms crossed into the neuronal RNAi sensitive TU3401 strain following RNAi by feeding on HT115 bacteria expressing dsRNA of the snx-1, snx-6 and snx-27. EV: Empty Vector control (light grey), Statistical significance was determined from the mean values of three independent experiments using ANOVA in conjunction with a Tukey's post-hoc multiple comparisons test (alpha = 0.05).

Supplementary Figure 1

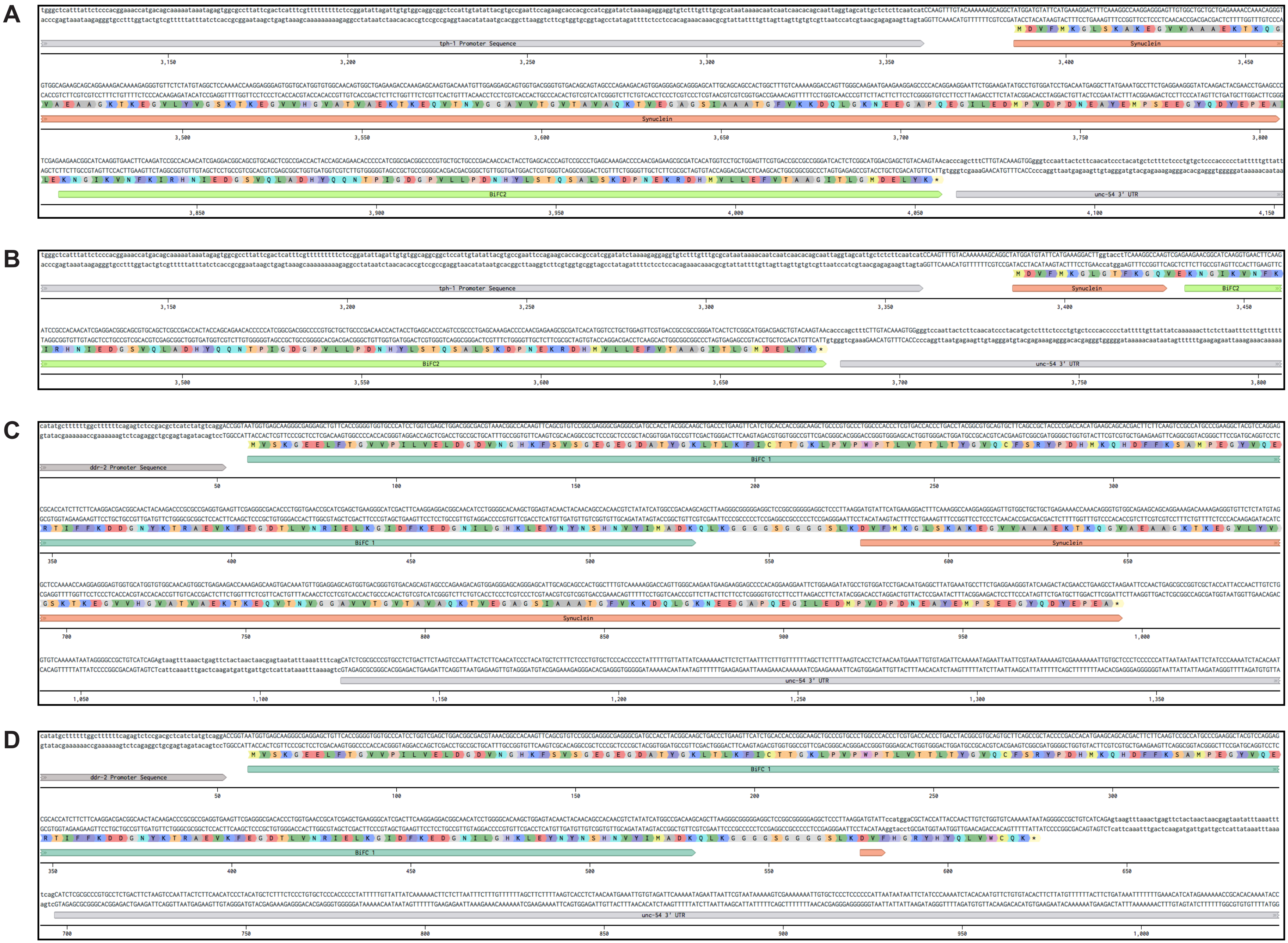

Supplementary Figure 2

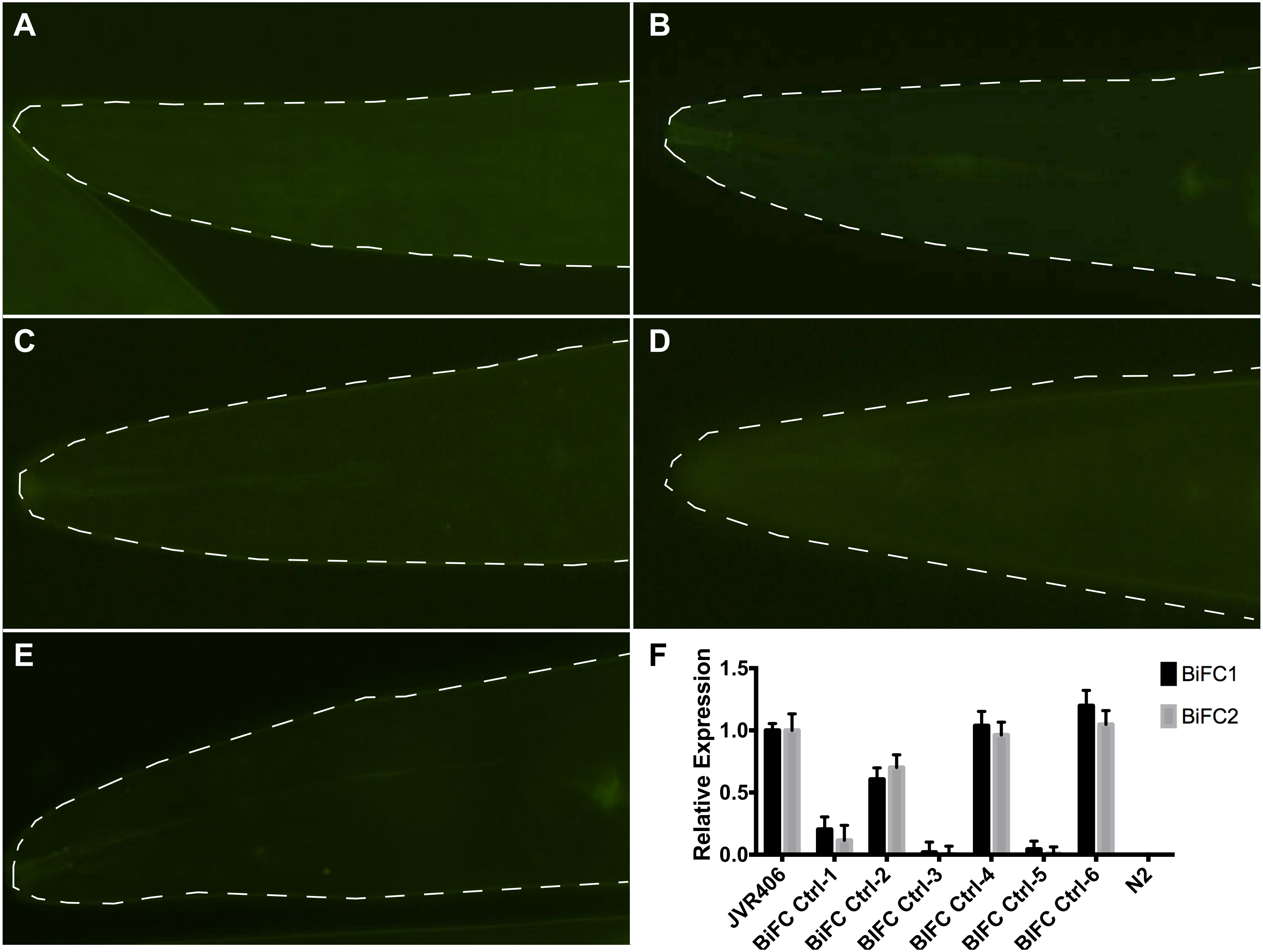

Supplementary Figure 3

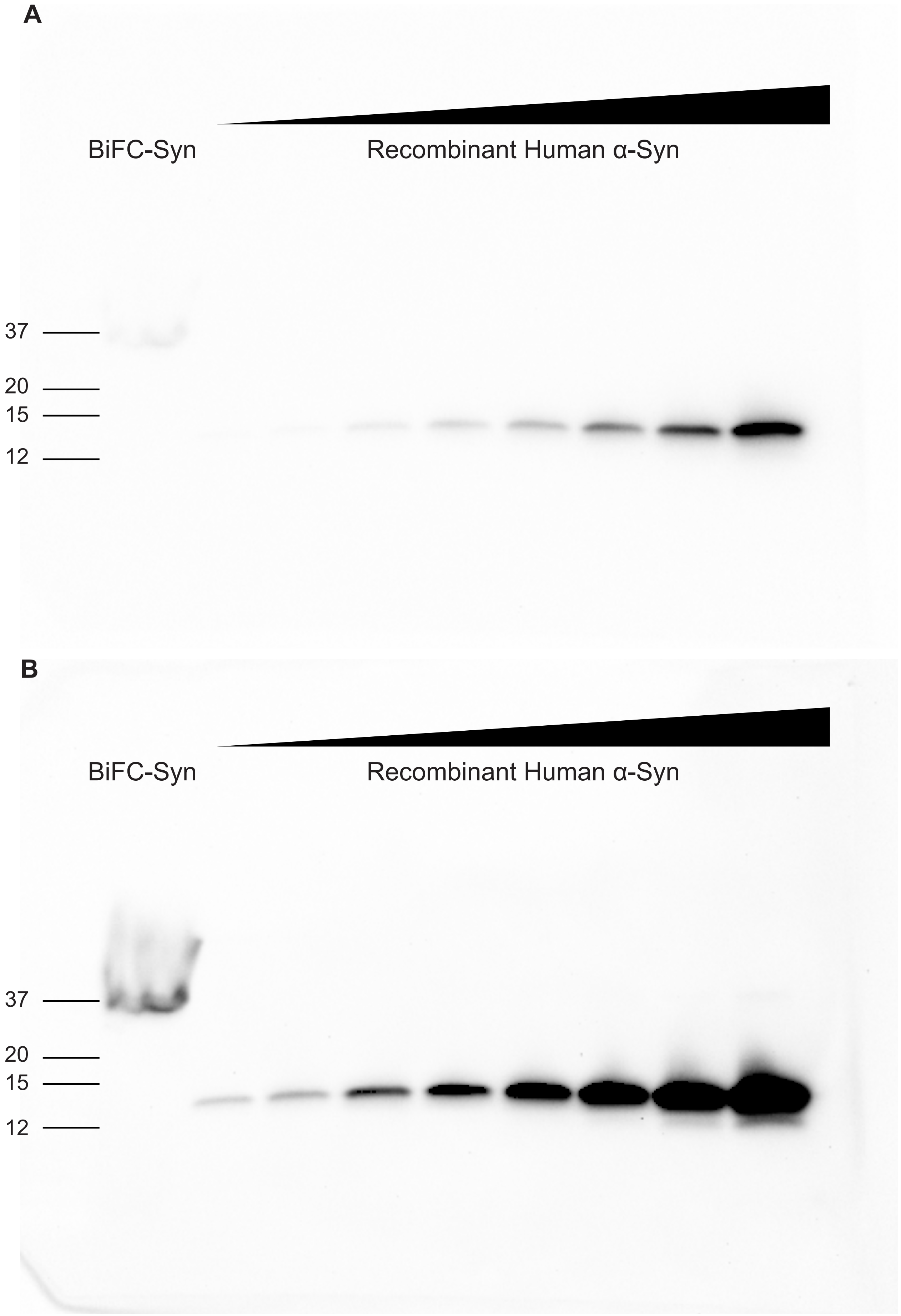

Supplementary Figure 4

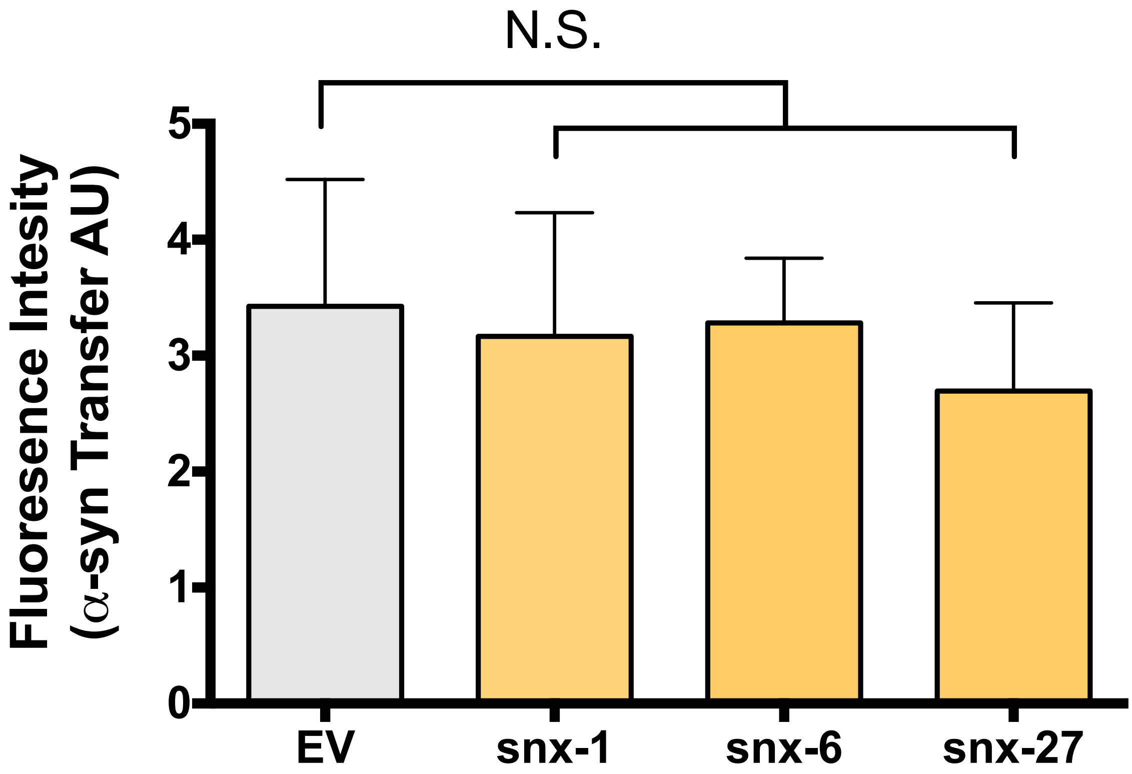

Supplement: Supplementary file 1 — Supplementary Information [file 41598_2017_7383_MOESM1_ESM.pdf]
